# Tumor Infiltrating Neutrophils Are Frequently Found in Adenocarcinomas of the Biliary Tract and Their Precursor Lesions with Possible Impact on Prognosis

**DOI:** 10.3390/jpm11030233

**Published:** 2021-03-23

**Authors:** Vittorio Branchi, Benedict Jürgensen, Laura Esser, Maria Gonzalez-Carmona, Tobias J. Weismüller, Christian P. Strassburg, Jonas Henn, Alexander Semaan, Philipp Lingohr, Steffen Manekeller, Glen Kristiansen, Jörg C. Kalff, Marieta I. Toma, Hanno Matthaei

**Affiliations:** 1 Department of General, Visceral, Thoracic and Vascular Surgery, University Hospital Bonn, 53127 Bonn, Germany; vittorio.branchi@ukbonn.de (V.B.); benedict.juergensen@ukbonn.de (B.J.); jonas.henn@ukbonn.de (J.H.); alexander.semaan@ukbonn.de (A.S.); philipp.lingohr@ukbonn.de (P.L.); steffen.manekeller@ukbonn.de (S.M.); joerg.kalff@ukbonn.de (J.C.K.); 2 Institute of Pathology, University Hospital Bonn, 53127 Bonn, Germany; laura.esser@ukbonn.de (L.E.); glen.kristiansen@ukbonn.de (G.K.); marieta.toma@ukbonn.de (M.I.T.); 3 Department of Internal Medicine I, University Hospital Bonn, 53127 Bonn, Germany; maria.gonzalez-carmona@ukbonn.de (M.G.-C.); tobias.weismueller@ukbonn.de (T.J.W.); christian.strassburg@ukbonn.de (C.P.S.)

**Keywords:** cholangiocarcinoma, biliary tract cancer, prognosis, neutrophils

## Abstract

Biliary tract cancer (BTC) is characterized by an intense stromal reaction and a complex landscape of infiltrating immune cells. Evidence is emerging that tumor-infiltrating neutrophils (TINs) have an impact on carcinogenesis and tumor progression. TINs have also been associated with outcomes in various solid malignant tumors but their possible clinical role in BTC is largely unknown. Tissue samples from patients with sporadic BTC (“spBTC” cohort, *N* = 53) and BTC in association with primary sclerosing cholangitis (“PSC-BTC” cohort, *N* = 7) were collected. Furthermore, tissue samples from 27 patients with PSC who underwent liver transplantation (“PSC-LTX” cohort) were investigated. All specimens were assessed for TIN density in invasive and precancerous lesions (biliary intraepithelial neoplasia, BilIN). Most spBTC showed low TIN density (LD, 61%). High TIN density (HD) was detected in 16% of the tumors, whereas 23% were classified as intermediate density (ID); the majority of both HD and ID groups were in T1–T2 tumors (83% and 100%, *p* = 0.012). TIN density in BilIN lesions did not significantly differ among the three groups. The HD group had a mean overall survival (OS) of 53.5 months, whereas the mean OS in the LD and ID groups was significantly shorter (LD 29.5 months vs. ID 24.6 months, log-rank *p* < 0.05). The results of this study underline the possible prognostic relevance of TINs in BTC and stress the complexity of the immune cell landscape in BTC. The prognostic relevance of TINs suggests a key regulator role in inflammation and immune landscape in BTC.

## 1. Introduction

Biliary tract cancers (BTC) are a heterogeneous group of malignancies arising from the epithelial cells of the intra- and extrahepatic biliary ductal system and gallbladder. These tumors account for approximately 3% of all gastrointestinal cancers and represent the second most common primary liver tumor [1,2]. Hence, BTC are rare, while in particular the incidence of intrahepatic cholangiocarcinoma is on the rise in Western countries [3]. BTC is one of the most aggressive cancer entities and radical surgery represents the only curative option [4]. However, only about 30% of patients with BTC are resectable at the time of first presentation [5]. Even after radical surgery, the median overall survival barely reaches three years [6].

Several risk factors for the development of BTC have been identified, such as chronic inflammation through primary sclerosing cholangitis (PSC), viral hepatitis, liver cirrhosis, and liver fluke [7]. In fact, BTC is histologically characterized by an intense stromal reaction while the stroma is densely populated by cancer-associated fibroblasts (CAF) and various infiltrating immune cells such as monocytes and tumor-associated macrophages (TAM). These inflammatory cells likely play a predominant role in tumor development and progression [8,9]. Thus, a biomarker function of easily obtainable systemic inflammation markers such as C-reactive protein (CRP), neutrophils to lymphocytes ratio (NLR), and platelets to lymphocytes ratio (PLR) has been intensely investigated in BTC in the recent years with remarkable success [10,11,12,13,14]. Neutrophils are the most abundant white blood cell subtype, thereby representing a crucial component of the innate immune system. While the main function of neutrophils is to fight infections by phagocytosis and elimination of microorganisms, a chronic neutrophilic inflammation has been associated with the early phase of various epithelial cancers. Interestingly, evidence is emerging that tumor-infiltrating neutrophils (TINs) are impacting progression and spread in later phases of the disease. Apparently, malignant tumors have the ability to induce myelopoiesis and to attract neutrophils to the tumor microenvironment. Here, these cells gather a protumorigenic, immunosuppressive phenotype, whereas the exact mechanisms and possible targeted therapies are currently under investigation [15]. Not surprisingly, TINs have been associated with outcome in various solid tumors [16,17]. However, their presence and possible prognostic role in BTC is still largely unknown and focus of the present study.

## 2. Methods

### 2.1. Patients and Tissues

For this monocentric retrospective study, data from three different patient cohorts were collected and analyzed.

#### 2.1.1. Patients with Surgically Resected Sporadic Biliary Tract Cancer (spBTC Cohort)

Tissue samples from 53 patients who underwent surgical resection for sporadic BTC (spBTC) between 2013 and 2018 at the Department of Surgery, Bonn University Hospital, were collected. Only patients without previous radiochemotherapy were selected. All patients were operated on in the absence of clinical signs of an acute infection. Patient serum was obtained shortly before surgery and analyzed for carcinoembryonic antigen (CEA), carbohydrate antigen 19.9 (CA19.9), and C reactive protein (CRP). Therapy options for every patient were discussed in our weekly interdisciplinary tumor board and all patients included were offered surgical resection. Demographic and clinical data, including age, gender, postoperative complications, hospital stay, and adjuvant chemotherapy were retrieved from the patients’ records. Tumor samples from the resected specimens were fixed in formalin and embedded in paraffin (FFPE) according to a standardized protocol. Survival data were available for all patients. The usage of archived diagnostic left-over tissues for tissue microarray (TMA) manufacturing, the analysis for research purposes, and patient data analysis were approved by the ethics committee, Bonn University Hospital (IRB number: 417/17). The study was carried out in compliance with the Helsinki Declaration.

#### 2.1.2. Patients with Primary Sclerosing Cholangitis and BTC (PSC-BTC Cohort)

Tissue samples from 7 patients with primary sclerosing cholangitis and BTC (PSC-BTC) were retrieved from archived diagnostic left-over FFPE tissue. All patients were operated on between 2010 and 2017 at the Department of Surgery, Bonn University Hospital. All tumor samples were obtained for diagnostic reasons during radical tumor resection or explorative laparotomy and open biopsy. A survival analysis was not performed in this cohort due to the small number of patients.

#### 2.1.3. Patients with PSC Who Underwent Liver Transplantation (PSC-LTX Cohort)

Tissue samples from 27 patients with PSC who underwent liver transplantation between 2003 and 2017 at the Department of Surgery, Bonn University Hospital, were collected from FFPE left-over tissue. A mean of 12 samples per patient, each from different localizations along the biliary ductal system, were analyzed. A survival analysis was not performed in this cohort due to the small number of patients.

### 2.2. Tissue Microarray Construction and Tumor-Infiltrating Neutrophil (TIN) Density Score Analysis in Sporadic BTC

For spBTC, a tissue microarray (TMA) was constructed according to standardized protocols. Briefly, four to six different 1 mm cores were taken from every tissue sample. For internal controls, normal kidney and normal liver tissue cores were included in each TMA block. A 2 µm section was stained following a standard hematoxylin and eosin staining protocol. Neutrophils were counted in each core by an experienced pathologist, who was blinded to tumor stage and patient characteristics. Apoptotic neutrophils, as well as neutrophils found inside the lumen of blood vessels were excluded. Then, the mean value was calculated for each tumor, as well as the 60 and the 85 percentile which we used as cut-offs. The use of these cut-offs was arbitrary in order to classified samples into “low density” if the TIN density score was lower than the 60th percentile (LD, score 0), “intermediate density”, if the TIN density score was between the 60th and 85th percentiles (ID, score 1), or “high density” if the TIN density score was higher than the 85th percentile (HD, score 2).

### 2.3. Neutrophils Density Analysis in Biliary Intraepithelial Neoplasia (BilIN)

For spBTC and PSC-BTC cohorts, diagnostic slides were reviewed for the presence of high-grade biliary intraepithelial neoplasia (BilIN) in the tumor proximity. For the PSC patients with PSC who underwent liver transplantation (PSC-LTX) cohort, all diagnostic slides were reviewed for the presence of high-grade BilIN in bile ducts. In the presence of multiple BilIN lesions, one representative high-grade BilIN was randomly selected. The total number of neutrophils in six 40× microscopic fields (three intraepithelial/intratumoral fields and three in periepithelial stroma) were assessed. Neutrophils that were identified in blood vessels were not counted. The mean value for each localization was then calculated.

### 2.4. BTC and BilIN Classification

All tumors were reclassified according to the UICC (Union for International Cancer Control) TNM classification system by the International Union Against Cancer, 8th Edition [18]. BilINs were classified according to established histopathological characteristics such as degree of cellular and structural atypia as recommended [19,20].

### 2.5. Statistical Analysis

Statistical analysis was performed in the R environment (RStudio Version 1.3, package survminer version 0.4.8) and SPSS Statistics Version 22 (IBM, Armonk, New York, NY, USA) [21,22]. Continuous variables are shown as mean or median with interquartile range (IQR). Univariate analysis was performed, and Kaplan–Meier (KM) plots were generated for overall survival (OS). KM curves were compared using the log-rank test. Multivariate analyses for OS were performed using the Cox regression method. Variables which were significant in the univariate analysis were included in the Cox regression model. Mean OS was indicated if the median OS was not reached. Comparisons between groups were made with Fisher’s exact test or Anova test, as appropriate. Pearson’s correlation analysis for TIN density in BilIN was performed and displayed as scatter plot. Statistical significance was assumed at a *p*-value < 0.05.

## 3. Results

### 3.1. Patient Characteristics

A total of 53 patients (29 females and 24 males, median age 67, range 38–81) were enrolled in the spBTC cohort. A majority of tumors was diagnosed as intrahepatic cholangiocarcinoma (IHC, *N* = 19, 36%), followed by distal cholangiocarcinoma (DC, *N* = 14, 26%), and perihilar cholangiocarcinoma (PHC, *N* = 13, 24%). A gallbladder carcinoma (GBCA) was diagnosed in seven patients (13%). Median preoperative CA19.9 and CEA levels were 53.6 kU/L (IQR 26.9–359 kU/L) and 2.2 ng/mL (IQR 1.2–3.0 ng/mL), respectively. Median preoperative CRP level was 17.2 mg/L (IQR 7.2–59.5 mg/L). Most primary tumors were classified pT2 (*N* = 19, 36%) or pT3 (*N* = 19, 36%), whereas 28% of the patients were staged pT1 (*N* = 15, 28%). A positive nodal status was found in 57% of the patients (N1, *N* = 30). Four patients (7%) with apparently resectable disease had to be classified M1 according to the final pathology report due to peritoneal carcinomatosis. Most tumors were moderately differentiated (G2, *N* = 31, 58%). Postoperative adjuvant chemotherapy was administered in a majority of patients (*N* = 33, 62%). Characteristics of the spBTC cohort are summarized in Table 1.

### 3.2. Correlation of TIN Density with Clinicopathologic Parameters

**Figure 1:**
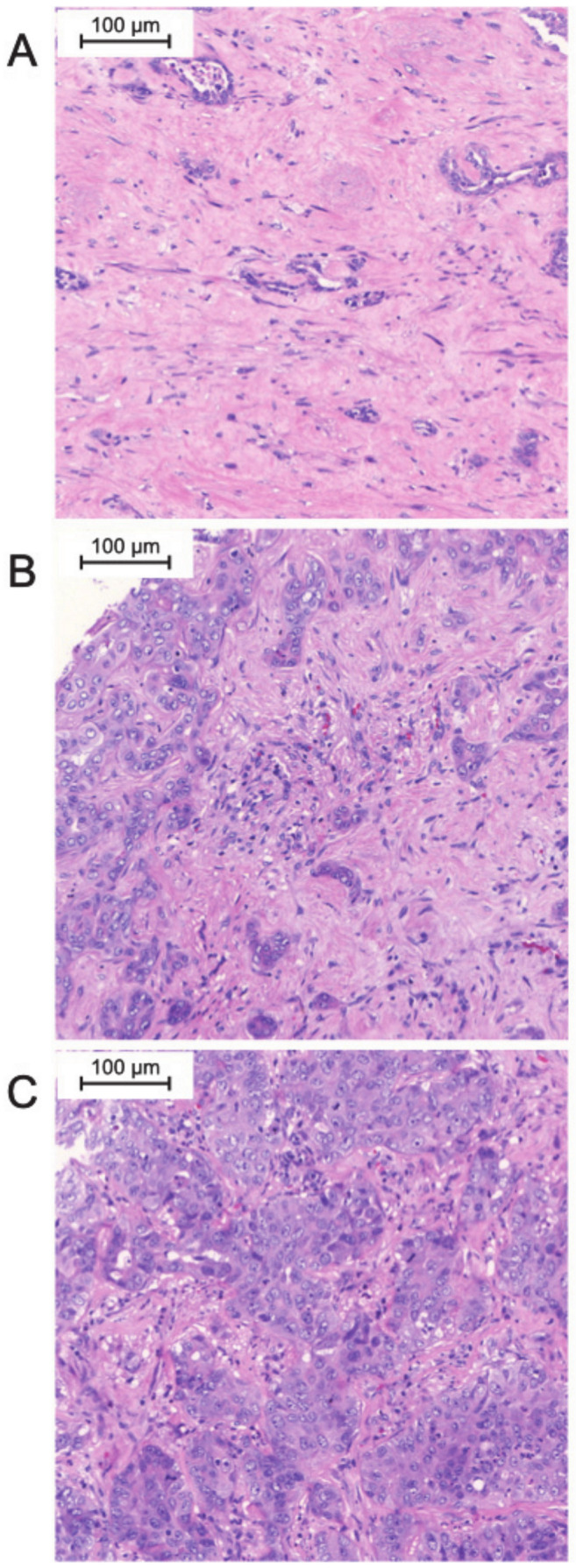
Examples of high-resolution microscopy images of biliary tract cancer with low (**A**), intermediate (**B**), and high (**C**) TIN density (hematoxylin and eosin staining).

Among the tumor samples, 16% were classified as tumors with a high TIN density (*N* = 8), while 23% were classified as intermediate (*N* = 12), and 61% were classified as low TIN-density tumors (*N* = 31). Two samples had to be excluded from the analysis because of tissue fragmentation during TMA preparation. Representative images of tumor sections with different TIN densities are displayed in Figure 1.

The demographics of the three groups were similar regarding age (mean age 67 in the LD group vs. 66 in the ID group vs. 67 in the HD group) and gender (58% females and 42% males in the LD group vs. 42% females and 58% males in the ID group and 50% females vs. 50% males in the HD group). Tumor stage distribution differed significantly in the three groups, since in the LD group the most represented T stage was T3 (*N* = 17, 55%). In the ID group, the majority of the patients had T1–T2 tumors (*N* = 10, 83%) and 17% (*N* = 2), also in the HD group, all had a comparatively smaller T1–T2 primary tumor (*p* = 0.012). The rate of N+ tumors was also significantly different in the three groups. In the LD group, 71% (*N* = 22) had a positive nodal status as compared with 25% (*N* = 3) in the ID group and 50% (*N* = 4) in the HD group (*p* = 0.025). No difference was observed regarding preoperative CA19.9, CEA, CRP, tumor location, M stage, grading, as well as lymphovascular and perineural invasion. Most of the tumors in the LD were IHC (*N* = 10, 32%) followed by DC (*N* = 8, 26%), GBC (*N* = 7, 23%), and PHC (*N* = 6, 19%). In the ID group, IHC and DC were the most represented tumor localizations (*N* = 5, 42%), followed by PHC (*N* = 2, 17%). In the HD group, IHC were the most frequent tumors (*N* = 4, 50%), followed by PHC *(N* = 3, 37%) and DC (*N* = 1, 12%) The LD group was the only group with samples from GBCA patients. The abovementioned varying TIN densities according to T stage and positive nodal status were mirrored in the respective UICC stages. In fact, in the LD group, most patients had advanced UICC stages III or IV, in the ID group the majority of the patients had earlier UICC stages I or II, and in the HD group 50% had a UICC stage I or II (*p* = 0.010). Clinicopathological characteristics of the three groups are summarized in Table 1.

### 3.3. Correlation of TIN Density with Outcome

**Figure 2:**
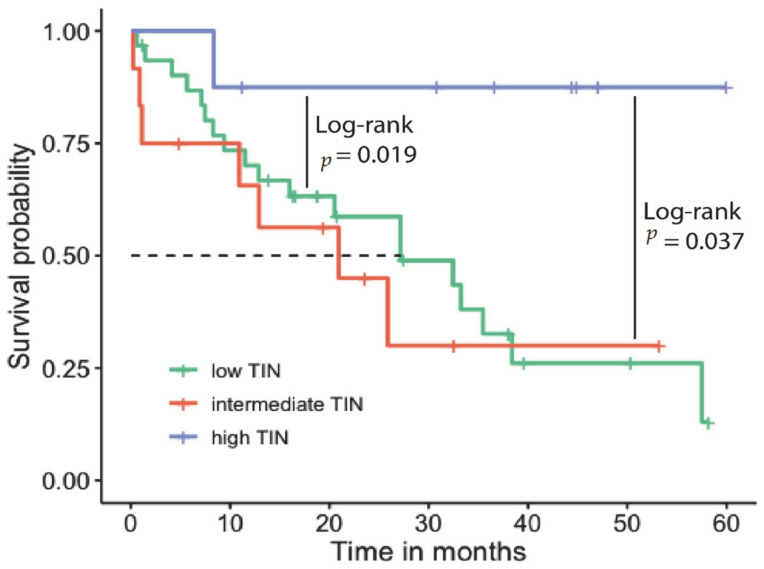
Kaplan–Meier (KM) plot showing survival probability stratified for tumor infiltrating neutrophiles (TIN) density. From the log-rank, only significant *p*-values are displayed.

Median follow-up time was 19.4 months (range 0.2–60 months). The HD group had a mean OS of 53.5 months (standard error (SE) 6.0, 95% confidence interval (CI) 41.7–65.4), whereas the mean OS in the LD and ID groups was significantly shorter (LD group, 29.5 months, SE 3.9, 95% CI 21.7–37.3, log-rank *p* < 0.05 and ID group, 24.6 months, SE 6.0, 95% CI 11.8–37.4, log-rank *p* < 0.05). The Kaplan–Meier plot in Figure 2 displays OS probabilities stratified for neutrophil density.

In the univariate survival analysis, a correlation with poor prognosis in HD vs. ID (hazard ratio (HR) 10.10, 95% confidence interval (CI) 1.22–83.50, *p* = 0.032) was found. When comparing HD vs. LD, a similar correlation with prognosis was found (HR 7.66, 95% CI 1.02–57.53, *p* = 0.048). In addition, univariate analysis showed that a UICC stage III was significantly associated with poorer prognosis as compared with UICC stage I (HR 4.48, 95% CI 1.01–19.92, *p* = 0.049). The results of the univariate analyses are summarized in Table 2. The results of the multivariate analysis are summarized in Supplementary Table S1.

### 3.4. Neutrophil Infiltration in BTC-Associated BilIN

**Figure 3:**
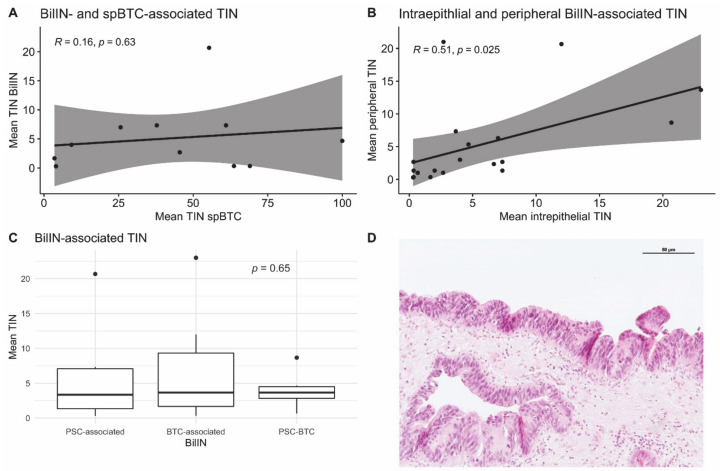
Scatter plot showing the relationship between tumor-infiltrating neutrophils (TINs) in spBTC and BTC-associated biliary intraepithelial neoplasia (BilIN) (**A**) and between intraepithelial and peripheral (stromal) TINs in BilIN lesions (**B**). Best-fitting lines and 95% confidence intervals as well as R coefficients and *p* values are displayed. Boxplots displaying mean infiltrating neutrophils (TINs) in BilIN from patients with primary sclerosing cholangitis (PSC), in sporadic BTC-associated BilIN and in PSC-related, BTC-associated BilIN (PSC-BTC). The lower and upper hinges correspond to the 25th and 75th percentiles. The upper/lower whiskers represent the largest/smallest observation less/greater than or equal to upper/lower hinge +/− 1.5 times the interquartile range (**C**). Exemplary section of a high-grade BilIN lesion from a patient with BTC. (hematoxylin/eosin staining) (**D**).

In the spBTC cohort, 23% of the patients (*N* = 12) had at least one high-grade BilIN lesion in the analyzed slides displaying their primary adenocarcinoma. In the PSC-BTC cohort, 57% of the patients (*N* = 4) had at least one high-grade BilIN lesion near the tumor. In the PSC-LTX cohort, at least one BilIN lesion was found in 25% of the explanted liver samples (*N* = 7). The mean TINs in the BilIN lesions from the cohort of patients with PSC who underwent LTX did not significantly differ from the mean TINs of BilIN lesions adjacent to sporadic BTC and PSC-associated BTC (7.0, standard deviation (SD) 8.1 vs. 4.9, SD 5.7 vs. 4.0, SD 2.5, *p* > 0.05) (Figure 3).

A positive correlation between intraepithelial TIN and stromal TIN was observed (Pearson’s *R* = 0.51, *p* = 0.025). The number of TINs in spBTC and PSC-BTC lesions did not correlate with the number of neutrophils infiltrating adjacent BilIN lesions (Pearson’s R = 0.16, *p* = 0.63). Patient characteristics of the PSC-LTX and PSC-BTC cohorts are summarized in the Supplementary Table S2.

## 4. Discussion

Neutrophils are a crucial component of the human innate immunity and the first circulating cellular responders in the case of acute tissue damage and infections. In cancer, neutrophils play a pivotal role in the tumor microenvironment and can acquire an antitumor (N1) or a protumor (N2) phenotype [23].

Several soluble factors produced by tumor cells promote TIN recruitment. Among others, CXCL1, CXCL2, CXCL5, CXCL6, IL17, IL 8, and CCL3 have been found to act as potent TIN chemoattractants [23]. TIN recruitment is followed by a polarization in either N1 or N2 phenotype due to a complex cytokine stimulation promoted directly from tumor cells and other tumor microenvironment cells [24]. Transforming growth factor-β (TGF-β) and interferon-β (IFN-β) have been found to be one of the most important promoters of neutrophil polarization in mice [25,26]. Therefore, some studies have addressed the possible use of TGF-β inhibition as a therapeutic approach in cancer [26]. However, the N1/2 polarization as well as the role of TINs in human cancers are still controversial [27].

Inflammation and inflammatory mediators are the hallmark of several risk factors associated with BTC [7]. In PSC, chronic inflammation of the bile ducts has been linked to the increased risk of BTC in this population [28]. Choledocholithiasis and cholecystolithiasis are characterized by cholestasis and chronic inflammation and are also considered risk factors for BTC [29]. Liver fluke infections, viral infections, and cirrhosis are similarly linked to cholangiocarcinogenesis [28].

Neutrophil infiltration has been evidenced and comprehensively investigated in various solid tumors revealing heterogeneous results. As such, high neutrophil infiltration proved to be related to chemosensitivity and longer recurrence-free survival in high-grade ovarian cancer [30]. Furthermore, high levels of intratumoral granulocytes negatively correlates with cancer-specific survival in patients with clear cell renal cell carcinoma [31]. In hepatocellular carcinoma, neutrophil infiltration has been shown to be a negative prognosticator after curative resection, correlating with angiogenesis progression and tumor recurrence in this entity [32,33,34]. Ino et al. described similar results in patients with resected adenocarcinomas of the pancreas [35]. The role of TINs has also been investigated in BTC. Gu et al. found that presence of TINs was significantly associated with adverse OS in a cohort of intrahepatic cholangiocarcinomas [36]. Another study found that a high density of CD15 (a carbohydrate epitope expressed on neutrophils) positive neutrophils correlated with shorter OS in cholangiocarcinoma [37]. In our cohort, we observed that patients with tumors with higher neutrophil infiltration had a better prognosis as compared with those with tumors of low or intermediate neutrophil infiltration. Our results regarding OS in resected BTC revealed intriguing aspects. On the one hand, tumors with the highest infiltration showed a longer OS as compared with the group with intermediate infiltration. On the other hand, the group with intermediate TIN infiltration had a tendency of worse OS as compared with the group with low TIN infiltration. However, in the group with high TIN infiltration, there were only T1 and T2 tumors. Moreover, the group with low TINs had a significantly higher N+ tumors as compared with the other groups. This could represent an important confounding factor, and therefore the results of the survival analysis should be interpreted with caution. A multivariate survival analysis of a larger cohort with matched groups could address this topic, provide more robust results, and reduce the bias. The different prognosis among the groups could also be explained based on the complexity of the tumor immune microenvironment in BTC, which includes tumor-associated fibroblasts, tumor-associated macrophages (TAM), dendritic cells, natural killer cells, and myeloid-derived suppressor cells. All these cells contribute to both tumor immunosurveillance and immunosuppressive functions. The tumors with high TINs in our cohort may represent a subgroup with a high immunological antitumoral activity. In fact, previous studies have reported that tumor-associated neutrophils could have a dual role. On the one hand, in early tumors, neutrophils could play a role in stimulating T-cell response [38]. On the other hand, neutrophils have an immunosuppressive activity in advanced tumors [39]. In our cohort, the tumors with high TINs were T1 and T2 tumors, whereas in the group with intermediate TIN, T1 to T3 stage tumors were represented. However, this could confirm previous observations about the dual role of TINs in cancer. Interestingly, the number of infiltrating neutrophils in BilINs did not significantly differ from those adjacent to sporadic BTC and those found in patients with PSC with or without BTC. Therefore, it is reasonable to think that neutrophils play a role in the development of a subset of BTC from BilIN towards invasive carcinoma, thus, underlying their tumor-promoting activity in early stage, possibly independent of predisposition.

Our study has some limitations. Firstly, our monocentric cohort was too small to build a proper validation of our preliminary findings which we anticipate in the future. In particular, due to the small BTC cohort and the small number of samples per group, a definitive conclusion about the prognostic role of TINs cannot be reached. A multicentric research effort could potentially address this limitation. Moreover, a multivariate survival analysis of matched groups could help by reducing the bias deriving from unbalanced groups. The retrospective design of this study carries intrinsic limitations, which should be taken into account when interpreting the results. In addition, our study did not focus on further TIN characterization. It is already known that infiltrating neutrophils have different phenotypes and capabilities, which lead to heterogeneous inflammatory responses [40]. This topic has not yet been deeply investigated in BTC and represents an intriguing research field with promising translational implications. Nonetheless, the phenotypical characterization of TINs was not the aim of the present study which mainly focused on prognostic implication. Furthermore, we enrolled patients with BTC irrespective of anatomical localization. This may be relevant when assessing prognostic factors for this tumor. However, some authors have described similar outcome for patients with BTC from different localizations but with comparable pathological characteristics [41]. BTCs are believed to be a stem cell-based disease and there are probably several different BTC stem cell populations involved in carcinogenesis [42,43]. In addition, there is growing evidence of a heterogeneous genetic landscape in BTC [44]. Due to the rareness and similar clinical behavior, we should find a prognostic factor that could be useful to the entire population of BTC patients, despite the proven heterogeneity of BTC.

## 5. Conclusions

In summary, the results of this study underline the frequency and possible prognostic relevance of TINs in BTC. Our clinically apparent, though somewhat ambivalent, results stress the complexity of the immune cell landscape in this fatal cancer entity that deserves further scientific dedication. The role of TINs as a prognostic factor in BTC remains unclear. The prognostic relevance of TINs should be further investigated in order to determine if neutrophils may act as a key regulator of inflammation and immune status in BTC. Further studies addressing the role of TINs would hopefully provide new insights to elucidate the associated tumor microenvironment for clinical implications in BTC.

## Figures and Tables

**Table 1:** Patient’s characteristics according to the extent of tumor-infiltrating neutrophils (TINs) in resected biliary tract cancer (BTC).

			TIN Density			
		**All Patients** ***N*** **= 53**	**Low** ***N*** **= 31**	**Intermediate** ***N*** **= 12**	**High** ***N*** **= 8**	
		*n*, mean, median (%, SD, or IQR)	*n*, mean, median (%, SD, or IQR)	*n*, mean, median (%, SD, or IQR)	*n*, mean, median (%, SD, or IQR)	*p*
Sex	W M	29 (54.7) 24 (45.3)	18 (58.1) 13 (41.9)	5 (41.7) 7 (58.3)	4 (50.0) 4 (50.0)	0.624
Age	≤67 >67	28 (52.8) 25 (47.2)	16 (51.6) 15 (48.4)	7 (58.3) 5 (41.7)	4 (50.0) 4 (50.0)	0.925
CA19-9		53.6 (26.9–359.0)	53.6 (28.3–160.4)	61.8 (20.3–360.4)	36.4 (29.7–1164.6)	0.325
CEA		2.2 (1.2–3.0)	2.4 (1.4–3.3)	2.2 (1.1–4.6)	1.2 (0.9–1.3)	0.080
CRP		17.2 (7.2–59.5)	24.7 (9.50–75.50)	11.0 (4.9–31.2)	19.4 (6.2–26.7)	0.396
Localization	IHC PHC DC GBC	19 (35.8) 13 (24.5) 14 (26.4) 7 (13.2)	10 (32.3) 6 (19.4) 8 (25.8) 7 (22.6)	5 (41.7) 2 (16.7) 5 (41.7) 0 (0.0)	4 (50.0) 3 (37.5) 1 (12.5) 0 (0.0)	0.312
T	T1 T2 T3 T4	15 (28.3) 19 (35.8) 19 (35.8) 0 (0)	6 (19.4) 8 (25.8) 17 (54.8) 0 (0.0)	4 (33.3) 6 (50.0) 2 (16.7) 0 (0.0)	4 (50.0) 4 (50.0) 0 (0.0) 0 (0.0)	*0.012*
N	N0 N1	23 (43.4) 30 (56.6)	9 (29.3) 22 (71.0)	9 (75.0) 3 (25.0)	4 (50) 4 (50)	*0.025*
M	M0 M1	49 (92.5) 4 (7.5)	27 (87.1) 4 (12.9)	12 (100.0) 0 (0.0)	8 (100.0) 0 (0.0)	0.462
L	L0 L1	39 (73.6) 14 (26.4)	22 (71.0) 9 (29.0)	9 (75.0) 3 (25.0)	7 (87.5) 1 (12.5)	0.743
V	V0 V1	44 (83.0) 9 (17.0)	24 (77.4) 7 (22.6)	11 (91.7) 1 (8.3)	7 (87.5) 1 (12.5)	0.671
Pn	Pn0 Pn1	24 (45.3) 29 (54.7)	13 (41.9) 18 (58.9)	6 (50.0) 6 (50.0)	3 (37.5) 5 (62.5)	0.851
G	G1 G2 G3	3 (5.7) 31 (58.5) 19 (35.8)	2 (6.5) 18 (58.1) 11 (35.5)	1 (8.3) 8 (66.7) 3 (25.0)	0 (0.0) 3 (37.5) 5 (62.5)	0.517
R	R0 R+	42 (79.2) 11 (20.8)	25 (80.6) 6 (19.4)	11 (91.7) 1 (8.3)	5 (62.5) 3 (37.5)	0.285
Stage	Stage I Stage II Stage III Stage IV	9 (17.0) 19 (35.8) 20 (37.7) 5 (9.4)	1 (3.2) 12 (38.7) 14 (45.2) 4 (12.9)	4 (33.3) 6 (50.0) 1 (8.3) 1 (8.3)	3 (37.5) 1 (12.5) 4 (50.0) 0 (0.0)	*0.010*
Postoperative Complications	Yes No	28 (52.8) 25 (47.2)	16 (51.6) 15 (48.4)	6 (50.0) 6 (50.0)	5 (62.5) 3 (37.5)	0.837
Chemotherapy	Yes No	33 (62.3) 20 (37.7)	22 (71.0) 9 (29.0)	6 (50.0) 6 (50.0)	4 (50.0) 4 (50.0)	0.356

**Table 2:** Results of the univariate analysis.

Endpoint	Subgroup	HR	CI 95%	*p*
	Age > 67 vs. ≤67	1.17	0.55–2.47	0.678
	Female vs. Male	1.02	0.48–2.14	0.963
	CEA high vs. low	1.75	0.73–4.20	0.207
	CA19.9 high vs. low	1.78	0.82–3.84	0.145
	TIN intermediate vs. high	10.10	1.22–83.50	0.032
	TIN low vs. high	7.66	1.02–57.53	0.048
	Complications Yes vs. No	1.30	0.61–2.78	0.498
	G2 vs. G1	1.63	0.22–12.34	0.636
	G3 vs. G1	1.72	0.22–13.66	0.607
	T2 vs. T1	0.83	0.31–2.23	0.706
	T3 vs. T1	1.26	0.51–3.11	0.612
	N1 vs. N0	1.69	0.79–3.62	0.179
	M1 vs. M0	1.07	0.25–4.53	0.927
	Stage 2 vs. Stage 1	3.23	0.71–14.66	0.128
	Stage 3 vs. Stage 1	4.48	1.01–19.92	0.049
	Stage4 vs. Stage 1	3.15	0.44–22.51	0.254
	R1 vs. R0	1.30	0.52–3.25	0.579
	V1 vs. V0	1.74	0.70–4.34	0.234
	L1 vs. L0	0.82	0.33–2.05	0.676
	Pn1 vs. Pn0	1.39	0.65–2.95	0.394
	Chemotherapy Yes vs. No	0.62	0.29–1.31	0.208

## Data Availability

The data presented in this study are available upon reasonable request from the corresponding author.

## Supplementary Materials

The following are available online at https://www.mdpi.com/2075-4426/11/3/233/s1, Table S1: Patient characteristics of the PSC-LTX and PSC-BTC cohort, Table S2: Multivariate analysis.
