# Supplementary material for: Tumor Infiltrating Neutrophils Are Frequently Found in Adenocarcinomas of the Biliary Tract and Their Precursor Lesions with Possible Impact on Prognosis
Source: J Pers Med. 2021 Mar 23;11(3):233. doi: 10.3390/jpm11030233 (PMC8004909; doi:10.3390/jpm11030233)
Supplement: Supplementary file 1 [file jpm-11-00233-s001.pdf]

Supplementary Table S1. Results of the multivariate analysis (Overall survival, OS; Hazard ratio, HR; Confidence interval, CI).

Endpoint	Subgroup	HR	CI 95%	*p*
OS				
	Stage 2 vs 1	5.93	0.73–48.17	0.096
	Stage 3 vs 1	22.64	2.41–212.41	0.006
	Stage 4 vs 1	8.37	0.66–106.20	0.101
	TIN low vs high	10.27	1.28–82.69	0.029
	TIN intermediate vs high	40.01	3.73–428.97	0.002

Supplementary Table S2: Patient characteristics of the PSC-LTX and PSC-BTC cohort.

		PSC-LTX	PSC-BTC
		n, median (% or IQR)	n, median (% or IQR)
**N**		27	7
**Sex**	W	9 (33.3)	4 (57.1)
	M	18 (66.7)	3 (42.9)
**Age**		46.7 (39.5 - 51.8)	48.0 (43.9 - 54.6)
